# Microbial Consortia for Plant Protection against Diseases: More than the Sum of Its Parts

**DOI:** 10.3390/ijms241512227

**Published:** 2023-07-31

**Authors:** Tomasz Maciag, Edmund Kozieł, Piotr Rusin, Katarzyna Otulak-Kozieł, Sylwia Jafra, Robert Czajkowski

**Affiliations:** 1 Department of Botany, Institute of Biology, Warsaw University of Life Sciences—SGGW, Nowoursynowska Street 159, 02-776 Warsaw, Poland; 2 Division of Biological Plant Protection, Intercollegiate Faculty of Biotechnology UG and MUG, University of Gdansk, Antoniego Abrahama Street 58, 80-307 Gdansk, Poland; 3 Laboratory of Biologically Active Compounds, Intercollegiate Faculty of Biotechnology UG and MUG, University of Gdansk, Antoniego Abrahama Street 58, 80-307 Gdansk, Poland

**Keywords:** biocontrol, crop protection, biocontrol agents, biopesticides, plant diseases

## Abstract

Biological plant protection presents a promising and exciting alternative to chemical methods for safeguarding plants against the increasing threats posed by plant diseases. This approach revolves around the utilization of biological control agents (BCAs) to suppress the activity of significant plant pathogens. Microbial BCAs have the potential to effectively manage crop disease development by interacting with pathogens or plant hosts, thereby increasing their resistance. However, the current efficacy of biological methods remains unsatisfactory, creating new research opportunities for sustainable plant cultivation management. In this context, microbial consortia, comprising multiple microorganisms with diverse mechanisms of action, hold promise in terms of augmenting the magnitude and stability of the overall antipathogen effect. Despite scientific efforts to identify or construct microbial consortia that can aid in safeguarding vital crops, only a limited number of microbial consortia-based biocontrol formulations are currently available. Therefore, this article aims to present a complex analysis of the microbial consortia-based biocontrol status and explore potential future directions for biological plant protection research with new technological advancements.

## 1. Introduction

The ever-growing human population has led to an increase in food consumption, with plants serving as the primary food source worldwide. However, the combined effects of climate change and global fruit and vegetable trade have accelerated the spread of essential crop pathogens [1]. Therefore, to address these issues without further environmental degradation, it is crucial to explore effective and safe alternatives to chemical methods of crop protection against plant diseases [2]. Biocontrol, an approach involving methods that utilize natural interactions between organisms, offers a potential solution [3]. Extensive research has been conducted in this field, leading to multiple attempts to develop biopesticides to combat key plant pathogens [3]. However, despite the efforts of the scientific community and industry, the availability of biocontrol formulations remains limited, and their activity is often unsatisfactory [4]. Therefore, it is suggested that the combination of diverse strains of microorganisms with multidirectivemechanisms of disease suppression (which are, among others, antibiosis, competition, or induction of plant resistance) into artificial consortia can help enhance the biocontrol agents’ activity, especially in changing environmental conditions [5].

Microbial consortia can contain a diverse array of microorganisms that exhibit variations in their environmental preferences, such as soil type, host plant, different preferential sites of colonization, and activity against different pathogen species [6]. Although individual microbial strains may possess different modes of action, the amalgamation of multiple microorganisms within consortia can broaden the spectrum of their activities against a wide range of plant pathogens [7]. Additionally, the microorganisms present in biocontrol consortia can contribute to plant growth promotion and/or enhance the activity of the other microorganisms, further increasing the potential of such products [5].

Although meta-analysis has shown that the consortia activity is more significant in the greenhouse condition compared with field settings, the protective effect of the consortia remains more stable than that of single-strain inoculations [8]. Despite the promising potential of microbial consortia, the availability of biological control formulations based on microbial consortia on the market is currently limited [9]. This review aims to address the present situation of using artificial microbial consortia for plant protection against diseases, including the possible causes of the current situation. This study also delves into the mechanisms utilized by microorganisms for their activity, interactions within the consortium, and their influence on consortia activity. We conclude this study by highlighting future research perspectives in this field.

### Historical Perspective

**Figure 1:**
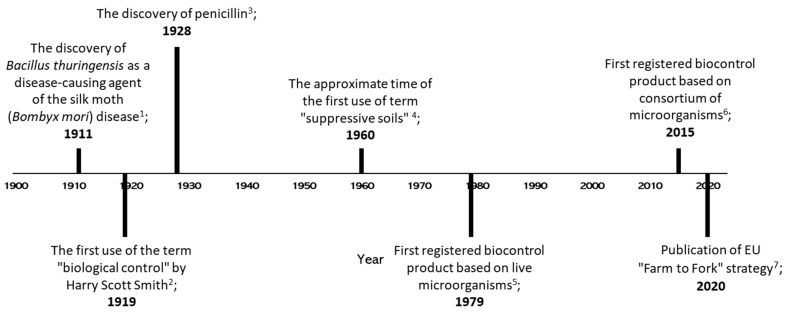
Timeline of events concerning biological control with consortia of microorganisms. 1—[9]; 2—[10]; 3—[14]; 4—[15]; 5—*Agrobacterium radiobacter* K84 against *A. tumefaciens* (Galltrol) [16]; 6—*Trichoderma asperellum* and *T. gamsii* against diseases caused by *Fusarium* spp., *Phytophthora* spp., Pythium spp., *Rhizoctonia* spp., *Sclerotinia* spp., *Sclerotium rolfsii*, *Thielaviopsis basicola*, *Verticillium* spp. (BIO-TAM 2.0) [4]; 7—publication of the “Farm to Fork” strategy as part of EU Green Deal to facilitate the implementation of environmentally friendly solutions for farming [17].

To comprehend the significance of biological control and evaluate the advantages and disadvantages of microbial consortia for plant protection, one must delve into the origins of human civilization, where plant health was attributed to soil’s visual quality. Even in modern agricultural practices, the organoleptic assessment of soil texture remains a reliable indicator of soil fertility [10]. Although the exact date of the first biological control application in practice is uncertain, Chinese farmers using ants against insect pests in storage are considered pioneers of this approach [11]. Similarly, the first identified microorganism used for biological plant protection was *Bacillus thuringiensis*, which has been used against insect pests such as the silk moth (*Bombyx mori*) [12]. Consequently, when Harry Scott Smith introduced the term “biological control”, it primarily referred to controlling insect plant pests [13]. During the XX century, scientists began elucidating the influence of the soil type on the probability and severity of certain crop diseases [14], although the terminology in this field remained inconsistent and ambiguous. Toward the end of the century, the term “suppressive soils”, describing soils that promote resistance against diseases, became widely used [15] (Figure 1). It was already recognized that microorganisms were responsible for this phenomenon and that this soil attribute could be transferred to new sterilized soil [16].

A few years later, the discovery of penicillin shook the scientific community [18]. Therefore, it seemed logical to explore the potential microorganisms in agriculture, particularly to search for potent antibiotic-producing strains against important plant pathogens. Indeed, this has led to the identification of numerous bacterial strains belonging to the *Pseudomonas* and *Bacillus* genera, renowned for their production of a wide array of antibiotics and other antimicrobials [19]. Therefore, the first biocontrol product (Galltrol) based on living microorganisms was developed, featuring *Agrobacterium radiobacter* K84, a producer of antibiotic agrocin 84 against *A. tumefaciens* [20]. However, the “silver-bullet” approach of finding a single strain capable of preventing multiple diseases on various crops in various soil types was bound to fail [21]. It was demonstrated that biocontrol strains’ activity was influenced by the environmental conditions in which they were deployed [22,23,24]. Therefore, the utilization of microbial mixtures with diverse modes of activity was proposed as a solution to overcome the challenges related to colonization under suboptimal conditions and enhance the stability of the protective effect of biocontrol products [5]. By the end of the XX century, the potential of consortia to address certain challenges in the biological control of pathogens had gained acceptance [25]. Nevertheless, the first biocontrol product containing a mixture of microorganisms was registered only in 2015 [4] (Figure 1). After that, a few more microbial consortia were registered for biological plant protection (Table 1).

The delay in the registration process of products based on multiple strains of microorganisms can be attributed to various challenges. These include difficulties in formulating and storing products containing living organisms, the slow technology transfer, and legislation prepared for chemical products [26]. However, there is a positive outlook for change in this scenario [27] thanks to scientific efforts aimed at improving such products’ performance and unraveling the source of unstable activity [28]. Additionally, the agricultural industry recognizes biological control as a potential source of novel “pesticides” that can be used in organic (green) farming [4]. A noticeable trend is the increasing ease and speed of registering microorganism-based products for agriculture, which paves the way for a wide range of microbial consortia-based products to enter the market [29,30,31]. An important advantage of pathogen biological control based on microorganisms, especially their mixtures, is their ability to protect plants from diseases and promote plant growth [32,33]. This opens up the possibility of registering a microbial consortium as a biofertilizer, which often follows a more straightforward product registration method [34]. Biofertilizers that protect plants from diseases can encourage farmers to adopt biological approaches, especially those interested in conventional, sustainable or organic farming [35].

**Table 1:** Biological control products available on the market are registered on the list of approved plant-protecting agents [36] (accessed on 5 May 2023). Formulations (Form.): WP—wettable powder; WG—wettable granules.

Active Substance	Trade Name	Distributor	Country	Form.	Target Crops	Target Disease
*Aureobasidium pullulans* DSM 14940 + DSM 14941	BLOSSOM PROTECT; BONI PROTECT; BOTECTOR	Bio-ferm Biotechnologische Entwicklung und Produktion GmbH	US; CA; EU; SK; TN; GB; NI; BE; DE; EL; ES; FR; HU; IT; LU; NL; PT; PL; RO; SI; SK	WP	Apple, medlar, pear, quince	Fire blight *Erwinia amylovora*
*Trichoderma virens* G-41 + *T. harzianum* Rifai T-22	RootShield^®^ PLUS WP	BioWorks, Inc.	US; CA	WG	Greenhouse and nursery vegetables, herbs, ornamentals, fruits, conifer tree seedlings, various trees, legumes, oil seeds, and peanuts	*Phytophthora,* *Rhizoctonia*, *Pythium, Fusarium, Thielaviopsis*, *Cylindrocladium*
*Trichoderma asperellum* ICC012 + T25 + TV1	XEDAVIR; PATRIOT GOLD; BIOTRIX; XEDAVIR PFNPE	Xeda International S.A.; Timac AGRO Espańa SA	IT; PT; FR; EU;	WP, WG	Greenhouse and open field vegetables	*Pythium* spp., *Phytophthora capsici, Rhizoctonia solani*
*Trichoderma atroviride* IMI 206040 + T11	Binab TF WP; Binab T Vector;	Borregaard Bioplant	SE; EU	WP	Tomatoes, strawberries, ornamental trees	*Botrytis cinerea*, *Chondrostereum* *purpureum*
*Trichoderma asperellum* ICC012 + *T. gamsii* ICC080	Tellus; Foretryx; Bio-Tam2.0; DonJon; Bioten WP; Blindar; Remedier	Syngenta; Isagro S.p.A.; Bayer; Gowan	NL; CA; PL; US; PT; FR; TN; CY	WP	Tomatoes, horticultural flowers, ornamental and tree crops	*Verticillium dahliae*, *Rhizoctonia solani*, *Sclerotinia sclerotiorum*, *Thielaviopsis basicola*, *Phytophthora capsici*
*Trichoderma asperellum* T25 + *T. atroviride* T11	Tusal	Newbiotechnic S.A.	FR; EL; GB; EU	WG	Strawberry, tomato, eggplant, pepper, cucumber, courgetti, melon, watermelon, pumpkin, cut flowers, lettuce, escarole, similars, trees, and shrubs	*Phytophthoracactorum*, *Rhizoctonia solani*, *Sclerotinia sclerotiorum*, *Phytophthora* spp., *Fusarium* spp., *Pythium* spp., *Phomopsis* sp.,

## 2. Ecological Interactions: Mechanisms of Plant Disease Control

**Figure 2:**
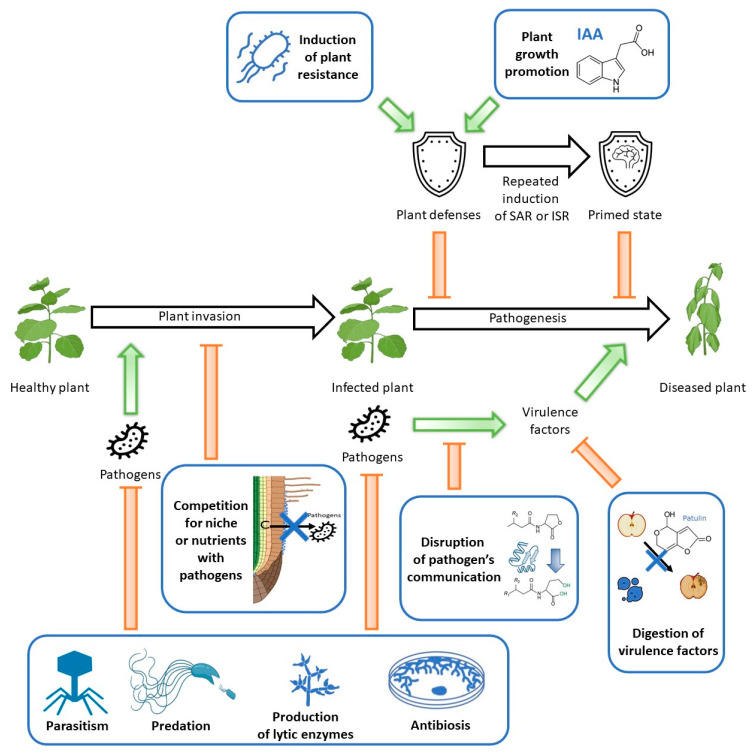
Possible mechanisms used by biological control agents (BCAs) (bolded and framed in blue) to prevent plant diseases. BCAs can directly protect plants from pathogen invasion by killing the pathogens before or during invasion by parasitism, predation, production of lytic enzymes, or antibiosis. They can also prevent or slow down pathogens’ invasion by blocking their ecological niche and/or competing for essential nutrients. The pathogen attack induces natural plant defenses, leading to systematic acquired resistance (SAR). These defenses can also be induced by nonpathogenic bacteria such as BCAs, leading to increased resistance through induced systemic resistance (ISR). It is also suggested that BCAs can increase plant resistance to pathogen attack by inducing plants’ general fitness via growth promotion through the inter alia production of plant hormones. Repeated induction of plant defenses, either by ISR or SAR, leads to the development of a state of increased resistance: a primed state. Pathogens that successfully invade plants coordinate the production of the virulence factors responsible for the development of the disease by a mechanism called quorum sensing. BCAs may disrupt this microbial communication through quorum quenching, which relies, among other things, on the digestion of signal molecules. BCAs can also disrupt pathogenesis via the digestion of virulence factors, thus preventing disease development. Red arrows demonstrate inhibition and green arrows represent induction.

Biological control agents (BCAs) have the ability to protect plants against diseases either by direct or indirect means. Direct protection involves the BCA acting on the disease-causing agent—a pathogen. This can be achieved via parasitism, predation antibiosis or production of lytic enzymes, and it can suppress pathogens before as well as during invasion. On the other hand, indirect activity alters the environment to decrease the presence of pathogens and the chance of disease development. This can be achieved through various mechanisms, such as inducing plant resistance or competition between the BCA and pathogens [36] (Figure 2). It is proposed that microorganisms can enhance plant resistance to pathogens by promoting plant growth, increasing the overall fitness of the plant, and decreasing the chance of disease development according to the disease triangle concept [26]. Biological control agents can also disrupt pathogenesis via the digestion of pathogens virulence factors or the disruption of their communication [37].

### 2.1. Induced Resistance

**Figure 3:**
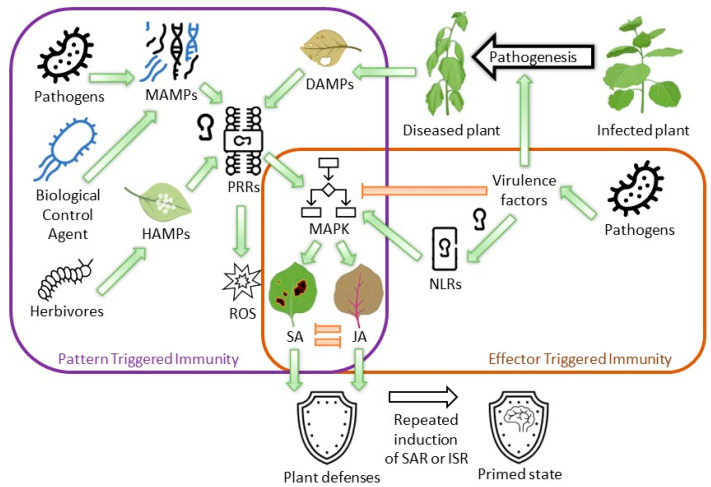
Pathways inducing plant defenses. Plants use pattern recognition receptors (PRRs) to recognize microbe-associated molecular patterns (MAMPs) and macromolecules by different microorganisms, both pathogenic and beneficial, for example, flagella. These receptors can also recognize antigens present due to the activity of herbivores’ herbivore-associated molecular patterns (HAMPs) or due to damaged plant tissues’ damage-associated molecular patterns (DAMPs). In response to these antigens, the plant releases reactive oxygen species (ROS) and activates mitogen-activated protein kinases (MAPKs). This mechanism of induced immunity due to the presence of these molecular patterns is called pattern-triggered immunity. Plants can also induce immunity in response to pathogen effectors—virulence factors that are recognized by nucleotide-binding domain leucine-rich repeat-containing receptors (NLRs)—and the immunity caused by the effectors is called effector-induced immunity. These two pathways act together to induce a plant’s immunity against plant pathogens through the salicylic acid pathway (against biotrophic pathogens) or the jasmonic acid pathway (against necrotrophic pathogens). These two pathways are antagonistic to each other. The repeated induction of plant defenses by these pathways, induced by the presence of pathogens by mechanisms (called systemic acquired resistance (SAR)) or plant beneficial microorganisms (called induced systemic resistance (ISR)), leads to the development of the state of prolonged increased resistance (primed state). Red arrows demonstrate inhibition and green arrows represent induction.

Throughout their evolution, plants have developed specific receptors, such as pattern recognition receptors (PRRs), that enable them to recognize various types of threats. These threats include the recognition of herbivore-associated molecular patterns (HAMPs) from herbivores, pathogen-associated molecular patterns (PAMPs) from pathogens, and microbe-associated molecular patterns (MAMPs) from other microorganisms; however, they can also recognize antigens present due to the breakage of plants’ physical barrier known as damage-associated molecular patterns (DAMPs) [38]. On recognition of the corresponding antigens, appropriate PRRs activate the PAMP-triggered immunity (PTI), triggering the release of reactive oxygen species (ROS), followed by the activation of mitogen-activated protein kinases (MAPK) (Figure 3) [38]. However, these patterns are often broad-range and can be produced by nonpathogenic bacteria, such as the conserved components of flagella that are found in different bacterial species. Therefore, to specifically recognize pathogen invasion, plants have developed effector-triggered immunity (ETI), which involves receptors recognizing effector proteins. These proteins are pathogen virulence factors and are recognized by internal receptors—nucleotide-binding domain leucine-rich repeat-containing receptors (NLRs) [39]. The crosstalk between these two pathways enables plants to mount appropriate responses against necrotrophic and biotrophic pathogens. For instance, against biotrophic pathogens such as *Pseudomonas syringae* [40], which invade living plant cells, plants induce the salicylic acid (SA)-dependent pathway of resistance, leading to hypersensitive response (HR) and local necrosis that stops the spread of the disease [41]. However, such a response would be inappropriate for nonpathogenic bacteria or microorganisms that have not yet breached plant cell walls [42]. Therefore, to combat necrotrophic pathogens such as *Pectobacterium carotovorum* [43] (which obtain their resources from disrupted or dying plant cells), plants activate jasmonic acid (JA)-dependent pathways, resulting in the accumulation of phenolic compounds, defensins, and cell wall strengthening to suppress the necrotrophic pathogen attacks [44]. Generally, JA- and SA-dependent pathways are antagonistic toward each other, although the exact interactions between those pathways are yet unknown [45]. The complexity of these interactions arises from the ongoing arms race between plants and their pathogens.

From the microorganism’s perspective, the plant immune response can be recognized as systemic acquired resistance (SAR) and induced systemic resistance (ISR). Although it is generally accepted that SAR is induced by pathogens through the SA-dependent pathway, nonpathogenic bacteria use the JA-dependent pathway to induce ISR—the term ISR refers to the induction of plant defenses by nonpathogenic microorganisms, regardless of the used pathway [46]. SAR is triggered by the presence of the pathogen and aims to reduce the likelihood of disease development on subsequent encounters with the pathogen. For example, when tobacco (*Nicotiana tabacum* L.) is infected by *Botrytis cinerea*, it develops resistance through a salicylic-mediated pathway, providing protection against subsequent pathogen attacks by *Pseudomonas syringae* and *B. cinerea* [47]. To achieve this, plants locally and systemically induce the expression of pathogen-related genes, facilitated by signaling through SA, the primary signal molecule in this mechanism [48]. However, it should be noted that the induction of SAR may require additional mechanisms to regulate its activation, depending on the specific plant [49]. This mechanism leads to the accumulation of reactive oxygen species (ROS) in the infected tissues [48], and it is typically triggered by the presence of pathogens [50]. Nonetheless, it has been demonstrated that nonpathogenic bacteria can also induce plant resistance through the SA-dependent pathway [51]. For example, *Pseudomonas aeruginosa* 7NSK2 induces resistance in tobacco against tobacco mosaic virus TMV through the SA-dependent pathway [52]. On the other hand, ISR is triggered by the presence of nonpathogenic microorganisms and aims to prime the plant for future encounters with pathogens [53]. Therefore, it is not surprising that ISR is widely employed to induce plant resistance by beneficial microorganisms [54]. For example, *Bacillus megaterium* L8 can protect cucumbers (*Cucumis sativus* L.) from seedling damping-off caused by *Pythium aphanidermatum* [55].

SAR and ISR are known to induce the expression of defense-related genes, and research has demonstrated that the induced resistance can persist and be inherited by subsequent generations [56]. This phenomenon is referred to as priming, which is facilitated by epigenetic changes, such as methylation alterations and histone modifications, that occur when plants are exposed to repetitive stress [57]. Consequently, primed plants exhibit a heightened ability to respond more swiftly to stressors [58]. Furthermore, it has been observed that plant-beneficial microorganisms can also trigger plant defenses, enabling the induction of a primed state without subjecting the plants to potentially harmful stresses [59]. This discovery opens up possibilities for employing priming as a mechanism for biological plant protection [60]. For example, when the tomato plants *Lycopersicon lycopersicum* (L. H. Karst.) are primed with *Pseudomonas fluorescens* N04 and *Paenibacillus alvei* T22, metabolic reprogramming occurs, resulting in enhanced resistance against *Phytophthora capsica* infections [61].

### 2.2. Competition

Microorganisms, however, have additional methods of safeguarding plants without relying solely on their natural defenses. Plant root exudates (organic metabolites secreted through roots) serve as a vital source of organic carbon for soil microorganisms [62]. As a result, the soil surrounding plant roots becomes a thriving hub for microbial abundance, diversity, and ecological interactions [63]. Microorganisms, to survive, compete with each other not only for space and primary nutrients but also for limited elements such as iron [64].

#### 2.2.1. Competition for Niches

Given that plant surfaces, including the rhizosphere, have finite space, the concept of microorganisms protecting plants through competition for niches has been proposed since the inception of biocontrol strategies [64]. The likelihood of such a mechanism is heightened by the fact that the root exudates are not uniform throughout plant development [65] and along the root [66]. Indeed, for the biological plant protection of fruits by yeast, the competition for niches and nutrients seems to be the most critical mode of action [67,68,69]. For example, the yeast *Rhodotorula mucilaginosa* reduces the colonization of apples by *Penicillium expansum* and *Botrytis cinerea* through rapid colonization and competition for available nutrients [70]. This mechanism is also utilized in the biocontrol of soilborne diseases [71,72]. This mechanism is reported inter alia to be used by nonpathogenic *Fusarium oxysporym* strains to protect tomatoes against *F. oxysporum* pathogenic strains [73] and eggplant against *Verticillium dahliae* [74]. Furthermore, it has been proven that biofilm formation plays a significant role in such mechanisms of activity, blocking the plant surface from pathogen invasion [75,76,77], which further suggests that the competition for niches plays a major role in biocontrol, although it is usually indistinguishable from the competition for nutrients [78].

#### 2.2.2. Competition for Nutrients

Competition among organisms typically revolves around the most limiting factor, which in the case of microorganisms in the soil, is often the availability of organic carbon [79]. However, in the natural environment, competition for nutrients is intertwined with competition for niches, and it can be distinguished by the use of a competition for nutrients assay [80,81], phenome microarray analysis [80,82], mutagenesis [83], and radioactive labeling [84]. Although competition for nutrients can play a vital role in the biocontrol of some soilborne diseases like *Pythium* damping-off [85] or *Fusarium* wilt [72], it is particularly significant in the biocontrol of postharvest fruit diseases, since carbon can be the most limiting factor on fruit surfaces [86]. In the case of *Pythium* damping-off, the selected active microorganisms did not produce any metabolites that directly suppressed the growth of *P. aphanidermatum*, and the growth suppression was associated with the concentration of glucose in the medium, correlating with cucumber protection against *P. aphanidermatum* [85]. In the rhizosphere, iron can be a more limiting nutrient for microorganisms.

#### 2.2.3. Competition for Iron

Despite the relative abundance of iron in the soil, it is predominantly inaccessible to plants and microorganisms, necessitating the evolution of iron mobilization strategies such as the use of iron chelators known as siderophores [87]. Microorganisms produce siderophores with varying iron-binding affinities and production costs, allowing them to adjust siderophore production in response to external conditions and competition [88]. Interestingly, many microorganisms produce multiple types of siderophores. This phenomenon is not merely a genetic extravagance but is crucial for the precise regulation of iron uptake [89]. One well-known example are the fluorescent *Pseudomonas*, which produce a potent siderophore pyoverdine, along with other genes responsible for synthesizing additional iron chelators in their genomes. These microorganisms have been extensively studied for their application in biocontrol [90]. Multiple fluorescent *Pseudomonas* species utilize this mechanism in biocontrol applications [91,92,93]. However, several studies have reported the direct antimicrobial action of siderophores toward bacteria and fungi [90,91,92,93], suggesting their activity in antibiosis, not competition “*sensu stricto*”. For example, *Pseudomonas donghuensis* produces two alternative iron chelators, pyoverdine and 7-hydroxytropolon [94]. Both of these iron chelators are essential for the antimicrobial activity of this species against different plant pathogens, although their production is influenced by iron and carbon availability [95].

### 2.3. Antibiosis

Microorganisms have additional mechanisms to gain a competitive advantage over their rivals through antibiosis, relying on other substances, such as organic acids, antibiotics, and bacteriocins [96]. Bacteriocins are antimicrobial peptides produced in ribosomes that usually target related microorganisms [97]. Although the study of bacteriocins for biocontrol is still limited, there is increasing interest in their potential application against antibiotic-resistant bacteria [98]. Bacteriocins can serve as effective antimicrobials in agricultural applications, particularly due to their narrow range of activity, which helps maintain a healthy microbiome of cultivated crops [99]. For example, *Bacillus subtilis* 14B produces Bac 14B bacteriocin, which contributes to the biological control properties of this strain against the crown gall-causing agents *Agrobacterium* spp. Production of antibiotics, in turn, is the most widely studied mechanism of action of biological control agents [100]. However, a precise definition of antibiotics is still lacking [101]. Scientific advancements in the field have led to the discovery of numerous antibiotic-producing microorganisms suitable for biological plant protection (Figure 1) [100]. *Agrobacterium radiobacter* K84, for example, produces the antibiotic agrocin 84 against *A. tumefaciens* in the first microorganism-based product for biological plant protection (Galltrol) [20]. The antibiotic concentration in the rhizosphere, although much lower than in artificial culture media, suggests that antibiotics in nature are not powerful bactericidal substances but rather suppress the growth rate and/or take part in communication between microorganisms [102]. However, these antibiotics play a crucial role for microorganisms in the soil, and their production is vital for biocontrol [103]. Given the increasing incidence of infections by antibiotic-resistant human pathogens, reducing the release of antibiotics and antibiotic-resistance genes into the environment has been proposed [104,105]. Therefore, exploring alternative modes of action can contribute to the safety of biological plant protection.

### 2.4. Production of Volatile Organic Compounds

Microorganisms have the capacity to produce volatile organic compounds (VOCs), providing versatile functions from antibiosis to communication. Although VOCs are not a functionally or structurally uniform group of compounds, their physical properties require a different study approach [106]. Many BCAs produce antimicrobial VOCs [107]. For instance, volatile compounds produced by *Pseudomonas fluorescens* WR-1 inhibit the growth of the important tomato wilt-causing agent *Ralstonia solanacearum* [108]. Interestingly, the activity of these volatile compounds extends beyond growth inhibition, as they can modulate *Ralstonia* metabolism to suppress virulence [108]. Furthermore, it has been observed that *R. solanacearum* could acquire resistance to the volatiles produced by *Bacillus amyloliquefaciens* T-5 but will lose the virulence factors responsible for its pathogenicity [109]. This phenomenon can alter the plant pathogen evolution toward decreased virulence. Additionally, BCAs can also promote plant growth through VOC production. For instance, *Bacillus amyloliquefaciens* not only produces volatile fungicidal compounds such as nonanone and 2-heptanone but also releases 2,3-butanediol and acetoin, which enhance the growth of *Arabidopsis thaliana* L. [110]. The diverse functions of VOCs make these compounds important players in the interactions between plant pathogens and beneficial bacteria, whose presence and impact should never be overlooked in the study of biological plant protection [107].

### 2.5. Production of Lytic Enzymes

The interaction between biocontrol agents and pathogens can exhibit distinct characteristics. Microbial biocontrol agents have the ability to produce chitinases, cellulases, proteases, and β-1,3-glucanases, which facilitate the breakdown of the cell wall components in plant pathogens, leading to cell lysis and leakage of nutrients from the cytoplasm [111]. The genus *Bacillus* is primarily known for its ability to produce and secrete a wide range of potent lytic enzymes, which can be used in biological plant protection and industrial applications [112]. Pre-cultivating biocontrol agents on chitin has been shown to stimulate chitinase production and enhance their performance [113]. The chitinase activity can also be induced in situ using natural microbiota by adding substrate (insect shells) to the soil to protect the plants from fungal diseases [114]. However, probably the best-known example of a microorganism that uses lytic enzymes for its biocontrol activity is the mycoparasitic genus *Trichoderma* [115].

### 2.6. Parasitism (Hyperparasitism)

Mycoparasitism refers to a specific type of parasitic interaction between fungi, wherein one fungal species senses, migrates, and envelops its prey to consume its resources [116]. This interaction is continuous, resembling typical parasitism. However, it often leads to the host’s death, differentiating it from classical parasitism but making it more applicable for biocontrol [117]. Although lytic enzymes are necessary for this interaction, parasitism represents a distinct mode of action for biocontrol agents [115]. Despite the potential of this mechanism for biological plant protection and numerous studies describing fungal hyperparasites [118,119], most of the research concerning the application of this mechanism concerns two genera: *Trichoderma* [120] and *Clonostachys* [121]. In the microbial world, bacteriophages can be considered as hyperparasites. Although they are not living organisms, their interaction with their host can be parasitic [117], and they seem promising for biological plant protection [122,123,124,125]. Bacteriophages are widely studied for their possible application in agriculture to target, for example, Soft Rot Pectobacteriacae [126]. In the case of bacteria, hyperparasitism, as a mode of action, is mainly used against plant pathogenic nematodes [127,128,129].

### 2.7. Predation

A fascinating and relatively unexplored mode of action of biological control agents can be predation. Bacterial predators are generally smaller in size compared to their prey and employ either a pack or single hunting strategy [130]. *Bdellovibrio* and *Bdellovibrio*-like bacteria are well-known examples of predatory microorganisms, although their prey range can vary [131,132]. Predation holds significant potential as a mode of action for biocontrol due to its inherent safety; however, currently, there are no commercially available products that utilize this mechanism. First, due to the difficulties of working with predatory bacteria (which need prey for growth), and second, due to the selection of microorganisms that are an appropriate prey range. If the bacteria have a prey range that is too narrow, they will quickly perish without a sufficient food source. On the other hand, an overly wide host range can result in limited or no beneficial effects when using such microorganisms [133].

### 2.8. Disruption of Pathogenesis

It is not always necessary to kill the pathogen to halt the progression of a disease. For example, the φ RSM filamentous phage can infect *Ralstonia solanacearum* and reduce its virulence via metabolic changes in the host, thus protecting the plant from wilting [134]. Additionally, microorganisms can degrade the virulence factors of the pathogens, for example, *Clavispora lusitaniae* 146 can degrade the *Penicillium digitatum* mycotoxin, patulin, and protect oranges, mandarins, tangerines, and grapefruits from fungal rot [135]. Pathogens rely on a process called quorum sensing to synchronize the production of their virulence factors [136,137]. However, other microorganisms can disrupt this communication by inhibiting the synthesis of signal molecules [138], enzymatic digestion [139], deactivation by cyclodextrin [140] or antibody binding [141], competition for receptor [142], or inhibition of the signal expression of genes activated by signal molecules [143,144]. Numerous biocontrol agents possess this potent yet relatively safe mode of action [37]. For example, *Ochrobactrum quorumnocens* uses AiiO hydrolase to degrade N-acyl homoserine lactones (AHLs) [145], therefore inhibiting the development of soft rot disease caused by *Pectobacterium parmentieri* SCC3193 in potato (*Solanum tuberosum* L.) [146].

## 3. Interactions between Components: Menace or a New Hope

**Figure 4:**
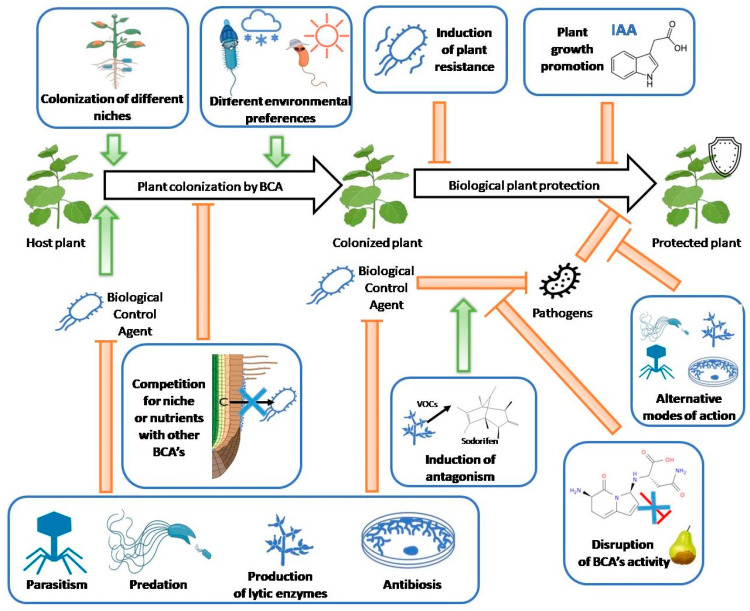
Possible mechanisms by which biological control agents can interact with other BCAs present in the applied consortium (bolded and framed in blue). BCAs can directly suppress other BCAs by killing them before or during colonization by parasitism, predation, production of lytic enzymes, or antibiosis. They can also prevent or slow down other components’ colonization by blocking their ecological niches and/or competing for essential nutrients. BCAs can also degrade the compounds responsible for other BCAs’ activity. On the other hand, use of multiple strains of BCAs has a positive effect of biocontrol activity thanks to the utilization of alternative modes of action, different environmental preferences and induction of BCAs’ secondary metabolism due to competitive conditions. Red arrows demonstrate inhibition and green arrows represent induction.

Even individual microbial strains can employ different modes of action to protect plants from diseases [147,148]. However, it has been proposed that using a mixture of bacteria can enhance the biocontrol effect in terms of not only its stability and the spectrum of application (in terms of the plant, soil type, and pathogen) but also its magnitude [149,150]. In nature, bacteria exist in complex, multispecies consortia with numerous interspecies and interkingdom interactions [63]. Therefore, employing multiple microorganisms as a consortium is expected to benefit their performance due to these interactions [5]. Therefore, this is why microbial consortia are commonly used as biofertilizers [151]. However, there are relatively few biocontrol products containing microbial consortia [34], not only due to the more problematic registration [152] of multiple-component-containing products but also to difficulties in the prediction of interactions between their components [153]. Microorganisms used for biocontrol usually produce a wide array of antimicrobial compounds, and the same modes of action used to fight the pathogens can negatively affect other consortium components [103]. For example, *Pseudomonas fluorescens* A506 degrades the antibiotics produced by strains *Pantoea vagans* C9-1 and *Pantoea agglomerans* Eh252, reducing their activity against the fire blight of pear [154]. This indicates incompatibility between the tested strains, highlighting the importance of confirming compatibility when composing consortia for biological plant protection. The same mechanisms used by biological control agents against plant pathogens (such as parasitism, predation, antibiosis, competition, and production of lytic enzymes but also digestion of substances responsible for their activity) can reduce the activity of other BCAs. On the other hand, BCAs can increase the protective effect with the use of alternative modes of action, different environmental preferences or by the induction of the secondary metabolism of other consortium components (Figure 4). There are various methods for assessing strain compatibility, each with its own advantages and disadvantages. However, the most commonly used approach to evaluate the biocompatibility of strains and their activity against selected pathogens relies on direct antagonism on artificial media [34]. Since microbial secondary metabolisms are highly dependent on the nutrients available [155], it has been suggested that strains for biological plant protection should be selected based on their in vivo rather than in vitro activity [156]. We believe that this principle should be applied to the selection and composition of microbial consortia, as the interactions within the consortium have a significant impact on its overall performance [5].

## 4. Successful Solutions

Table 1 provides an overview of the biocontrol products available on the market. Although knowledge transfer from science to industry may not occur rapidly, and various factors impact the selection of products on the shelves, it offers valuable insights into the potential for success. In the literature, numerous examples of complex consortia involving different microorganisms for combating various diseases can be found [5]. However, this diversity is not fully reflected in the range of biocontrol products registered for crop protection (Table 1). Farmers have access to multiple approved biological control products based on only a limited number of different microbial consortia. The challenges in registering products with multiple active ingredients contribute to this situation. Additionally, it is noteworthy that the current solutions for biocontrol predominantly rely on the use of *Trichoderma* spp. [157], despite the wide array of microorganisms available for such purposes.

*Trichoderma* is an extensively studied genus for biological plant protection, and numerous studies focus on identifying new isolates with promising biocontrol potential [158,159]. The popularity of this genus stems from the number of modes of action utilized by *Trichoderma* spp. [160] and the resulting potential to not only protect plants from important pathogens [161] but also to produce spores with a high survival rate during formulation [162]. Additionally, their ability to promote plant growth enables the use of *Trichoderma* strains as both biocontrol agents and biofertilizers [163]. We anticipate that this newly discovered *Trichoderma* species will quickly find their way onto the market of biocontrol products [164].

On the other hand, there are a wider range of species utilized in consortia for biofertilizers or biostimulants [165,166]. However, the legal status of these consortia, similar to biocontrol products, is in urgent need of revision [24,25,26,167]. Nevertheless, the relatively low number of biocontrol formulations can also be attributed to inadequate knowledge exchange between industry and academia [168,169]. Therefore, it is imperative to improve communication among scientists, plant protection product producers, farmers, and regulatory authorities. By enhancing collaboration, we can meet technological demands, address pressing agricultural challenges, and establish a safe and efficient environment for the registration of biocontrol products.

## 5. Future Research Perspectives

However, there are other crucial questions and issues related to biological plant protection that require our attention in further research to deepen our understanding of the subject. One key aspect that needs to be addressed is the interactions between biocontrol agents and pathogens. Although we have knowledge of the potential modes of action employed by biocontrol strains [36], most of the research dedicated to their study was performed in vitro only. Since the nutrient conditions on plants are very different from on artificial media [102], different mechanisms might be favored in the environment, and the known interactions (e.g., antibiotics production), although still important, may be different in nature [170].

Additionally, there is a need to study the interactions between BCAs and their hosts, especially since many strains used for biological plant protection rely on the induction of plant natural defenses [171]. Plants, being a significant source of organic carbon, play a crucial role in shaping the microbial environment by adjusting their root exudation based on their developmental stage [65]. Consequently, they can modulate microbial metabolism [172] and species composition [173,174]. We need to understand better the interactions between plants and their microbiome, and how they can modulate it to harness those interactions for agricultural production. Currently, the mechanism by which plants distinguish between beneficial and harmful microorganisms [175] remains a mystery. However, it has been suggested that plants can modulate their microbiome through the release of specific nutrients to enhance competitiveness [176]. Plant-beneficial microorganisms tend to produce and resist various antimicrobials [47], which gives them an advantage in competitive conditions [177].

There is a pressing need to investigate the antagonistic interactions between plant-beneficial strains since these interactions can have both positive and negative effects on their overall performance [5]. On the one hand, microbial strains can not only stimulate others to produce metabolites essential for their plant protective function [178] but can also outcompete them or diminish their activity [154]. This complexity adds challenges to the composition of microbial consortia for biological plant protection. Therefore, studying the interactions between highly antagonistic strains in biocontrol is crucial in order to inform the design of effective consortia.

An exciting avenue for such analysis could be the utilization of multi-omics data that are growing in number [179]. By integrating publicly available genomes, phenomes, transcriptomes, proteomes, and metabolomes in the meta-analysis, we can unravel general scientific experiment trends [180,181]. However, due to the vast amount of data present in publicly available databases and the increasing number of publications, traditional analysis methods are becoming increasingly challenging and call for the development of automated methods [182].

Although we anticipate an increase in the availability of biocontrol products using microbial consortia in the near future, the specific details of the registration process for such products remain uncertain. In addition, there is a clear requirement for improved communication of scientific findings to society to enhance knowledge transfer. Finally, the influence of meta-analysis is expected to grow, as it is necessary to effectively incorporate the vast amount of data published in this field.

## 6. Conclusions

Thanks to the efforts of the scientific community, numerous strains of microorganisms suitable for biological plant protection have been identified. However, despite the vast number and diversity of these strains, farmers still tend to prefer chemical methods. This preference can be attributed, in part, to the limited efficacy of microbial-based products and the challenges posed by the variability among different cropping systems. The suggested solution to this issue is the use microbial consortia, which can combine various microorganisms and different modes of action, increasing the stability of plant protection. Although there are a considerable number of promising microbial consortia isolated from various sources encompassing different modes of action, including growth promotion, the number of biocontrol products based on microbial consortia are minimal and contain only two genera, *Aureobasidium* and *Trichoderma*. The reason for this situation is that it is probably still difficult to register microbial-based plant protection products, especially those containing multiple species. To address this problem, we need to improve communication between academia, industry, administration, and the general public.

## Data Availability

Data sharing is not applicable to this article.
